# Organ-based characterization of B cells in patients with systemic lupus erythematosus

**DOI:** 10.3389/fimmu.2025.1509033

**Published:** 2025-01-23

**Authors:** Yunan Wang, Rui Zhao, Qian Liang, Shiwen Ni, Mei Yang, Liwei Qiu, Juan Ji, Zhifeng Gu, Chen Dong

**Affiliations:** Department of Rheumatology, Research Center of Clinical Medicine, Research Center of Clinical Immunology, Affiliated Hospital of Nantong University, Medical School of Nantong University, Nantong University, Nantong, China

**Keywords:** systemic lupus erythematosus, B cell, organ damage, organ specific features, therapy strategies

## Abstract

Systemic lupus erythematosus (SLE) is a chronic, inflammatory, and progressive autoimmune disease. The unclear pathogenesis, high heterogeneity, and prolonged course of the disease present significant challenges for effective clinical management of lupus patients. Dysregulation of the immune system and disruption of immune tolerance, particularly through the abnormal activation of B lymphocytes and the production of excessive autoantibodies, lead to widespread inflammation and tissue damage, resulting in multi-organ impairment. Currently, there is no systematic review that examines the specificity of B cell characteristics and pathogenic mechanisms across various organs. This paper reviews current research on B cells in lupus patients and summarizes the distinct characteristics of B cells in different organs. By integrating clinical manifestations of organ damage in patients with a focus on the organ-specific features of B cells, we provide a new perspective on enhancing the efficacy of lupus-targeted B cell therapy strategies.

## 1 Introduction

Systemic lupus erythematosus (SLE) is a systemic autoimmune disease that affects multiple organ systems. It is characterized by the abnormal activation of lymphocytes, disruption of immune tolerance, and production of autoantibodies (1–6). The high heterogeneity of the disease, its diverse features, and the alternating stages of onset and remission create significant challenges for effective management of lupus patients (4). Patients with lupus experience a range of issues, including sleep disorders, sexual dysfunction, fertility concerns, periodontal disease, and secondary osteoporosis (7–12). Additionally, anxiety, depression, and other emotional disorders in lupus patients relate to the abnormalities in lymphocyte function and inflammation (13–15). These adverse factors severely impact the prognosis and quality of life for lupus patients, sometimes leading to suicidal ideation and behavior (16). Therefore, gaining a comprehensive understanding of the pathogenesis of lupus is crucial.

An imbalance in immune systems, along with genetic factors, infections, and other influences, can trigger the onset of lupus. Various inflammatory mediators, dysregulated adaptive immune responses, and impaired immune tolerance contribute to abnormal cytokine secretion, as well as disrupted intracellular and intercellular signaling. These disruptions lead to impaired recruitment and activation of B lymphocytes, which ultimately participate in the onset and progression of lupus (17, 18). Autoreactive B cells produce excessive amounts of autoantibodies and pro-inflammatory cytokines, initiating an inflammatory cascade and immune response that ultimately induce organ damage in patients (19). Notably, these autoantibodies can emerge several years before the clinical symptoms of lupus appear (20, 21).

**Figure 1:**
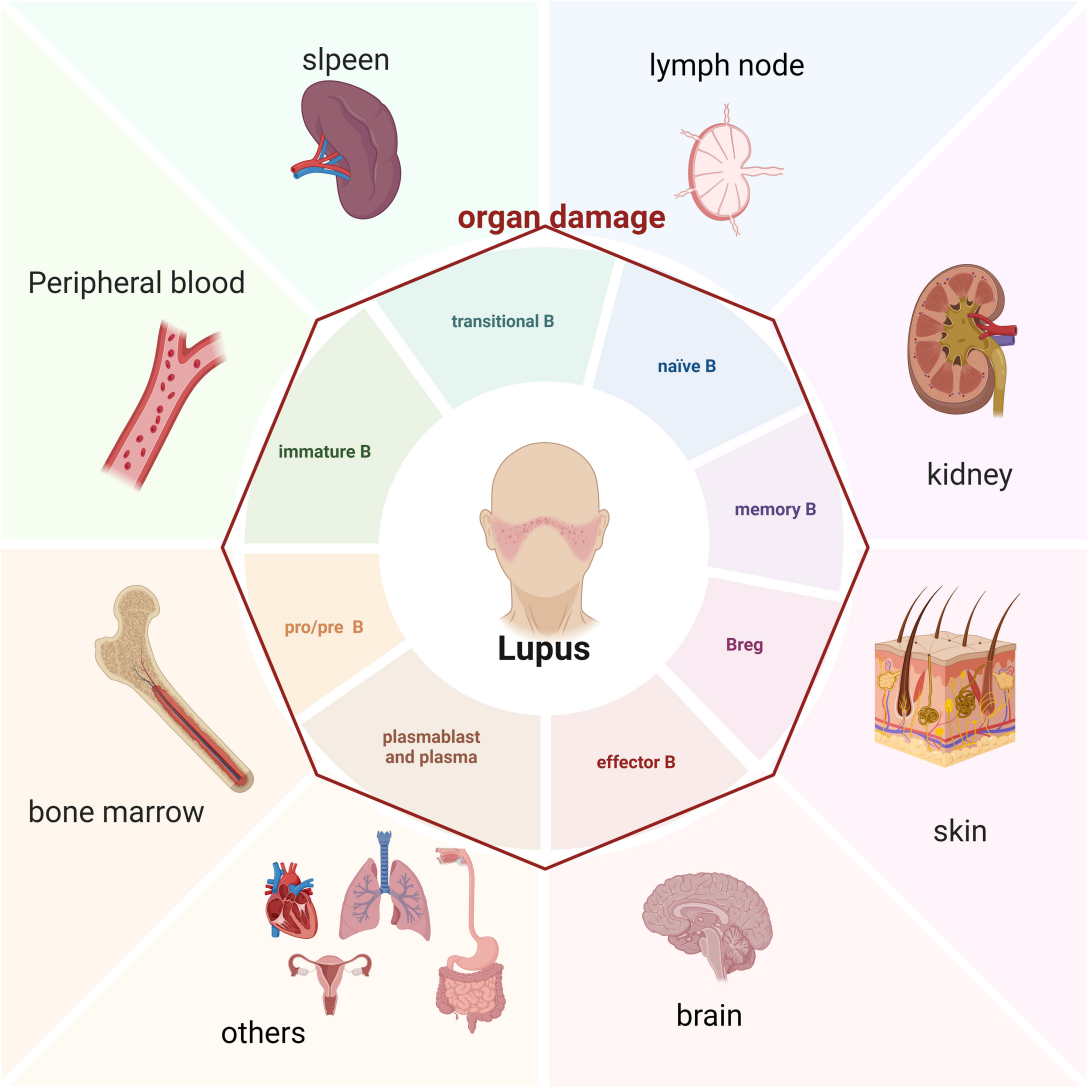
B cell and damaged organ in SLE patients (Created in BioRender. Dong, C. (2025) https://BioRender.com/s25g537).

Given the crucial role of B cells in lupus, scientists and medical experts have actively explored treatment strategies targeting these cells. Clinically, non-specific treatments such as glucocorticoids and immunosuppressive drugs are widely employed. In recent years, lupus patients have also received treatment with targeted biological agents related to B cells, including belimumab, anifrolumab, and telitacicept, as well as CAR-T therapy strategies (22–25). However, many patients do not benefit from these therapies. SLE is a systemic, diffuse autoimmune disease with variable multi-organ and multi-system involvement, and B cells may exhibit distinct phenotypes and functions in different tissues and organs (**Table 1**). Therefore, understanding B cell heterogeneity across organs and further elucidating the pathogenesis of lupus may unlock new therapeutic targets and improve treatment outcomes, which is essential for improving organ damage and prognosis in lupus patients. This review will focus on the development and differentiation pathways of B cells in lupus patients and their specific characteristics in different organs, providing evidence for potential therapies (**Figure 1**).

**Table 1:** B cell population changes in each tissue/organ.

Tissues/Organs	Subsets	Phenotypes	Tendency
Bone marrow	Pro-B	CD19^+^CD20^+^CD34^+^CD38^+^CD45R^+^	Reduced
	Pre-B	CD19^+^CD20^+^CD38^+^CD40^+^CD45R^+^	Reduced
	Immature B	CD19^+^CD20^+^CD40^+^CD45R^+^lgM^+^	Reduced
	Transitional B	CD10^+^CD19^+^CD20^+^CD24^hi^CD28^hi^CD27^-^	Increased
	Naive B	CD19^+^CD20^+^CD23^+^CD40^+^CD150^+^	Increased
	Memory B	CD19^+^CD20^+^CD27^+^CD40^+^CD150^-^CD38^-^	Increased
	LLPC	CD19^-^CD38^hi^CD138^+^	Increased
Blood	Breg	CD19^+^CD24^hi^CD27^+^	Reduced
	ABC	CD11c^hi^T-bet^+^	Increased
	UMBC	CD27^+^IgD^+^	Reduced
	DNB	IgD^-^CD27^-^	Increased
Spleen and lymph gland	B cell	CD19^+^ or B220^+^	Reduced
Kidney	LLPC	CD138^+^CD19^lo/-^ or CD138^+^B220^lo/-^	Increased
	ATM/ABC	CD24^-^CD20^hi^	Increased
Skin	UMBC	CD27^+^IgD^+^	Increased
	DNB	IgD^-^CD27^-^	Increased
Brain	Activated B	CD19^+^CXCR4^+^	Increased

LLPC, long-lived plasma cells, Breg, regulatory B cells, ABC, age-related B cells, UMBC, unswitched memory B cells, DNB, double negative B cells, ATM, atypical memory B cells.

## 2 Imbalance of B-cell immune tolerance of lupus patients

The adaptive immune system can recognize multiple pathogens and induce specific immune responses, thereby providing effective immune defense. During this process, adaptive immune cells, particularly B cells, may exhibit potential auto-reactivity. To manage this risk, the body has evolved mechanisms of immune tolerance to monitor auto-reactive cells, preventing them from becoming pathogenic effector cells and thereby avoiding autoimmune diseases such as lupus.

The control mechanisms for B cell development can be categorized into central immune tolerance and peripheral immune tolerance, depending on the stage and location of development. Central immune tolerance eliminates 50% to 75% of auto-reactive immature B cells generated in the bone marrow through processes such as clonal deletion, receptor modification, or receptor editing [the rearrangement of new light chains and their substitution on the original B-cell receptor (BCR)]. Tolerance is also induced on B cells by down modulation of BCRs (26). Despite this, a significant number of auto-reactive B cells (20% to 40%) escape central tolerance and migrate to the periphery, where they exist as transitional and naïve B cells (27). In the peripheral compartment, immune tolerance predominantly regulates the affinity maturation of auto-reactive B cells and eliminates those that strongly respond to self-antigens through clonal inactivation and clearance. Key mechanisms of peripheral immune tolerance include BCR signaling transduction, VDJ gene rearrangement, competitive inhibition of BAFF, and the induction of auto-reactive B cell migration to the periphery (28–30). Dysregulated BCR signaling initiates autoimmunity by modulating the naïve BCR repertoire during the development of immature and transitional B cells, and by promoting the activation of autoreactive B cell clones (31). VDJ gene usage is significantly biased of SLE patients, particularly in CD27^+^IgD^+^ unswitched memory B cells and plasmablast (32). The levels of BAFF are commonly overexpressed in SLE, which support the survival of autoreactive B cells and prevent their deletion. Overexpression of BAFF in mice may result in the development of an SLE-like disease, such as polyclonal B cell proliferation, production of multiple autoantibody, and increased circulation of immune complexes and renal immunoglobulin deposition (33).

Auto-reactive B cells may evade the monitoring mechanisms of central and early peripheral immune tolerance and subsequently enter the germinal center. Within the germinal center, IgG^+^ memory B cells can recover the quantity or type of cells previously eliminated during central immune tolerance through high-frequency somatic mutations and other processes. Non-auto-reactive B cells can also acquire self-reactivity, highlighting the crucial role of germinal center immune checkpoints in regulating auto-reactive plasma cells (34, 35). Numerous studies using animal models demonstrate that germinal center immune checkpoints function through various pathways, including follicular rejection, apoptosis mediated by *FAS*, inhibition via *FcyRIIB*, receptor modification, follicular dendritic cells, regulatory T cells, and follicular regulatory T cells (36–40). Furthermore, organized aggregates of immune cells formed in non-lymphoid tissues, such as tertiary lymphoid organs and other ectopic structures, may contribute to autoimmune diseases by failing to respond to tissue-specific auto-reactive B cells or even promoting their expansion (41, 42). The extra-follicular pathway represents another special pathogenic mechanism, which has been implicated in the pathogenesis of lupus in both mice and humans, particularly in the progression of LN (41, 43, 44). Thus, an imbalance in immune tolerance, including functional abnormalities in central, early peripheral, and late peripheral immune checkpoints, may lead to the expansion of auto-reactive B cells, resulting in the development of autoimmune diseases such as lupus.

The mechanisms underlying the disruption of immune tolerance remain unclear. Currently, researchers commonly use single-cell transcriptome sequencing, single-cell BCR sequencing, and flow cytometry with the 9G4 antibody to characterize lupus-specific autoantibodies encoded by the immunoglobulin heavy chain gene *VH4-34*. These methods help measure auto-reactivity and reflect the landscape of human B-cell immune tolerance. Healthy individuals rarely produce 9G4^+^ class-switched B cells; however, lupus patients with high disease activity exhibit significantly elevated levels of 9G4^+^ IgG in serum, along with an enrichment of *VH4-34* in antibody-secreting cells (45, 46). Furthermore, *VH4-34* accounts for a substantial proportion of naïve B cells (47), with levels reaching up to 2% to 10%. The 9G4 antibody can generate self-reactivity against dsDNA, apoptotic cells, and other self-antigens associated with lupus (48, 49). Studies indicate that somatic hypermutations can produce amino acid changes in the hydrophobic residues of *VH4-34* or enhance glycosylation to mask these hydrophobic residues, which may help reduce auto-reactivity. Although these mutations can diminish some auto-reactivity, *VH4-34* ^+^ B cell and plasma cell clones continue to undergo high-frequency amplification due to their strong auto-reactivity to various self-antigens. Thus, merely clearing *VH4-34* ^+^ B cells is insufficient to suppress lupus-specific self-reactivity (46, 48, 50). In healthy individuals, the germinal center effectively inhibits 9G4^+^ B cells, whereas lupus patients exhibit high expression levels of these cells, producing large quantities of autoantibodies. The failure to clear *VH4-34* alone underscores the impaired immune tolerance in lupus patients, making effective control of the disease particularly challenging (51).

## 3 B cells in different organs and tissues of lupus

### 3.1 Abnormal development of B cells in bone marrow may be the initiating factor for lupus

The development and differentiation of B cells begin in the bone marrow. Histopathological changes observed in the bone marrow, along with abnormal quantitative and qualitative features of hematopoietic stem cells, indicate that the bone marrow is one of the primary damaged organs in lupus patients (52, 53). In active lupus patients, CD34^+^ hematopoietic stem/progenitor cells are fewer than in healthy donors. These cells show high expression of apoptosis-related markers and tend to differentiate into bone marrow stromal cells (54, 55). Furthermore, the diversity of the BCR-H pool and the length of the BCR-H complementarity-determining region 3 (CDR3) in peripheral blood mononuclear cells from lupus patients are significantly reduced. Additionally, pre-selection BCR-H CDR3 sequences in lupus patients exhibit abnormal shortening, suggesting that the early development of bone marrow B cells and the BCR repertoire are compromised in these individuals (56).

Recent studies in mice have enhanced our understanding of how several pro-inflammatory cytokines, including interferon (IFN)-I, tumor necrosis factor (TNF)-α, and interleukin (IL)-6, inhibit the development and generation of lupus B cells (57–60). For instance, knocking out *EZH2* in B cells downregulates the key transcription factor *XBP1*, which impairs the differentiation of plasmablasts in MRL/lpr mice. This impairment reduces autoantibody production and improves the progression of glomerulonephritis (61). Additionally, the drug Ophiopogonin D can enhance disease progression in MRL/lpr mice by decreasing the number of CD19^+^ B cells in both bone marrow and peripheral circulation (62). However, due to the invasiveness of bone marrow puncture in humans, research on bone marrow B cells in lupus patients remains limited.

In a case report involving a lupus patient with progressive multifocal leukoencephalopathy, researchers observed a significant reduction in bone marrow B cells, along with mutations in the *GATA2* and *CDH7* genes (63). These genes are closely associated with the proliferation, differentiation, and functional maintenance of B cells. Palanichamy et al. reported that inhibition of early B cell development correlates strongly with elevated interferon levels in the bone marrow (58). Using a combination of single-cell RNA sequencing and BCR sequencing, Dong et al. revealed developmental disorders in early B cells (pro-B and pre-B) in lupus patients. These disorders are linked to increased disease activity, heightened interferon signaling, systemic and local inflammatory activation, oxidative phosphorylation, and other overactivated metabolic pathways. Notably, the abnormal features of bone marrow B cells can be reversed once the patient achieves clinical remission (64–66). Additionally, researchers noted elevated levels of atypical memory B cells and long-lived plasma cells in the bone marrow of lupus patients (64). Overall, existing research on the development, differentiation, pathogenesis, and targeted therapy of bone marrow B cells in lupus patients is relatively limited. Therefore, further in-depth investigations are essential.

### 3.2 Abnormal circulating B cells in lupus patients are associated with disease activity

Lupus patients exhibit abnormal manifestations of B cells, characterized by an imbalance in B cell subtypes. Notably, there is an increased number of memory B cells relative to immature B cells, alongside excessive BCR responses, heightened calcium influx due to receptor cross-linking, and increased tyrosine phosphorylation of downstream signaling molecules (67). Abnormal BCR signaling can increase the risk of lupus development: This enhanced signal transduction mediated by BCR can lower the activation threshold for peripheral B cells, leading to a disordered lupus B cell phenotype (68). The BCR signaling pathways (VAV2, PLC-γ2) is significantly upregulated, affecting calcium influx and cytoskeletal remodeling (69). Abnormal calcium influx mediated by BCR may interfer RAG expression and immunoglobulin receptor editing, which contributes to the persistence of autoreactive B cells and the progression of lupus (70). Additionally, B cells from lupus patients show elevated rates of somatic hypermutations and class switching recombination, which contribute to the pathogenicity of plasma cells (46, 71, 72). To identify various B cell phenotypes, researchers use flow cytometry based on surface markers. In clinical practice, flow cytometry is a key tool used in B cell research, which allows absolute and relative B cell counts, and enables the assessment of B cell phenotype and function at the single-cell level, while allowing the analysis of large cell populations (73). Studies have reported that certain B cell subsets, including regulatory B cells (Breg, defined as CD19^+^CD24^hi^CD27^+^)↓, plasma cells↑, CXCR5^-^ immature B cells↑, double-negative B cells (DNB, IgD^-^CD27^-^)↑, age-associated B cells (ABC, defined as CD11c^hi^T-bet^+^)↑, unswitched memory B cells (UMBC, CD27^+^IgD^+^)↓, CD19^+^Siglec-10^+^ B cells↑, and CD226^+^ B cells↑ in lupus patients (74–76). Notably, lupus patients have a reduced number of Breg cells, which demonstrate functional impairments. The proportion of Bregs is negatively correlated with SLE disease activity index (SLEDAI) score, but positively correlated with the level of serum C3 and C4 in patient with SLE (77). These cells exhibit decreased responses to CD40 stimulation and lower IL-10 secretion, creating barriers to inhibiting immune responses, maintaining immune tolerance, and restoring immune system homeostasis (18, 78). ABCs can persist in tissues and rapidly differentiate into antibody-secreting cells upon re-exposure to the antigens or innate stimuli. ABCs exhibit upregulation of B cell signaling, lipid and glucose metabolism, and endocytosis in lupus (79). The proportion of ABCs, CD19^+^Siglec-10^+^ B cells, and CD226^+^ B cells are positively correlated with SLEDAI score and anti-dsDNA titers, but negatively correlated with serum complement levels (75, 80). The number of CD27^+^IgD^+^ B cells is significantly reduced in SLE patients, with impaired production of natural antibody-like IgM and IL-10, and defective clearance of apoptotic cells. These cells correlate positively with white blood cell (WBC), blood platelet (PLT), and serum C3 levels, but negatively with serum creatinine levels, SLEDAI score, and anti-dsDNA titers (81).

The abnormal number and function of B cells in lupus are regulated by key factors such as BAFF, serum soluble B cell maturation antigen (BCMA), Toll-like receptors (TLRs), IL-6, and IL-21 (20, 82–86). Abnormal upregulation of recombinant activated genes in the peripheral blood B cells of lupus patients can lead to BCR mutations, resulting in the production of self-reactive B cells (87). A significant correlation exists between BCMA levels and various indicators of disease activity, including antinuclear antibody (ANA) titers, anti-dsDNA titers, immunoglobulin levels, and hematological parameters in lupus patients (88). BCMA may serve as a biomarker and a potential therapeutic target for SLE. BAFF promotes the survival, proliferation, antigen presentation, and differentiation of self-reactive B cells by binding to its receptors, including transmembrane activators and calcium modulators (TACI), BCMA, and other ligands (89). Belimumab, a therapeutic anti-BAFF monoclonal antibody, may demonstrate clinical efficacy in SLE patients through various mechanisms (31): belimumab binds to soluble BAFF and inhibits it from binding to its receptors (BAFF receptor, TACI, and BCMA), reducing activation of early B cells and differentiation into memory B cells and antibody-producing plasma cells (90, 91); belimumab significantly reduces expression of BCMA on the surface of immature B cells, non-class switched and class-switched memory B cells (92). TLRs play a crucial role in B cell activation; BCR-driven immune complex uptake stimulates TLR-7 and TLR-9, which further activates the type I interferon response in B cells and enhances the production of autoantibodies, contributing to the pathogenicity of lupus (93). TLR-7 is expressed in various B cell subtypes and causes chronic stimulation of endogenous RNA through an autophagy-dependent mechanism, leading to further upregulation of TLR7 and B cell activation (94, 95). In lupus patients, immature B cells are regulated by sensors of viral RNA such as TLR-7 and can differentiate into extra-follicular double-negative memory B cells (CXCR5^-^CD11c^+^). These cells exhibit high expression of interferon-related genes and undergo significant amplification, ultimately differentiating into plasma cells and contributing to the pathogenesis of lupus (96). TLR-9 signaling is essential for autoantibody production in mice and enhances the differentiation of B cells and plasma cells that produce autoantibodies in humans (97). TLRs are considered potential promising therapeutic targets in SLE. Using TLR antagonists or anti-TLR monoclonal antibodies to selectively prevent extracellular or endosomal TLR ligation has become an attractive treatment strategy for SLE, including TLR7 blockers (IRS661, DS-7011a), TLR7/8/9 antagonists (chloroquine), TLR7/8 blockers (Afimetoran, Enpatoran, E6742) and TLR2/4 inhibitors (vitamin D3) (98, 99). Additionally, abnormal interactions between T cells and B cells, such as shortened interaction times in the germinal center, can lead to insufficient signaling for self-reactive B cells, thereby increasing their survival rates (100).

### 3.3 Splenic and lymph node B cell abnormalities unlock new therapeutic targets for lupus

As secondary lymphoid organs, spleen and lymph nodes play a crucial role in regulating immune tolerance, particularly in the pathogenesis of lupus. Splenomegaly and lymphadenopathy are important indicators of pathological damage in SLE. It is reported that approximately 60% of SLE patients present with symptoms of localized or generalized lymphadenopathy at some stage of disease progression (101). Nakatani K et al. (102) found that the number of splenic B cells decreased in an age-dependent manner in MRL/lpr mice, with a reduced expression of their inhibitory receptors FcγRIIb1 and CD22. A functional defect of these receptors exacerbated autoimmune symptoms in mice models. The proportion of MZB1^+^ MZ B cells and MZB1^+^ plasma cells was increased both in lupus patients and lupus-prone (NZB x NZW)F (1) [BWF (1)] mice. MZB1, a B cell-specific and endoplasmic reticulum (ER)-localized protein, is a key participant in antibody secretion. MZB1 may enhance lupus disease progression by modulating Ca2^+^ homeostasis and IgM production of B cells (101). In imiquimod-induced lupus mouse model, imiquimod activated TLR7 receptor, activating downstream MyD88 and TRAF6, and releasing mitochondrial dsDNA and dsRNA through MyD88 and TRAF6 to further activate of MDA5 and cGAS pathways in splenic B cells (103). These intracellular signaling pathways activated B cells and increased autoantibody production.

In recent years, a series of diagnostic and therapeutic strategies of targeting spleen and lymph node B cells have been developed in lupus. TIGIT-Fc fusion protein suppressed autoantibody production through the regulation of SPI-B-PAX5-XBP1 axis-mediated B-cell differentiation. In MRL/lpr mice, TIGIT-Fc fusion protein decreased the proportion of B220^+^ B cells, plasmablasts, and plasma cells, while increased the proportion of naïve B cells. In cGVHD mice, the proportion and number of B cells were reduced, including plasmablasts, germinal center (GC) B cells, and memory B cells with the treatment of TIGIT-Fc fusion protein (104). AIM2 was highly expressed in B cells of lupus patients and mice models. AIM2 deficiency in B cells alleviated lupus symptoms and reduced the frequency of CD19^+^ cells in lymph nodes and spleen, as well as splenic GC B cells and plasmablasts/plasma cells of lymph node via Blimp1-Bcl-6 axis in pristane-induced lupus mice (105). Currently, researches on spleen and lymph node B cells of lupus mainly focus on animal models, and large clinical trials are still lacking, requiring more investigation in the further.

### 3.4 Local infiltration of B cells exacerbates renal tissue damage in lupus patients

The kidney is a vital organ affected by lupus, with approximately 50% of lupus patients developing LN, which can result in kidney failure or even death. The primary pathological feature of LN is the production of autoantibodies against nuclear and cellular antigens, leading to the deposition of immune complexes in the glomerulus. Glomerular situ immune complex is formed by secondary binding to nucleosomes from renal cells, leading to widespread acute renal damage and loss of nephron units. which ultimately results in chronic, irreversible kidney dysfunction (106). Additionally, the deposition of immune complexes activate the complement system, and complement factors can directly induce immune complex–related renal inflammation and immunopathology (107). Various subtypes of B cells have been characterized as playing significant roles in the onset and progression of LN. Notably, the proportion of IFNβ^+^ naïve B cells show a strong positive correlation with the incidence of LN and the deposition of immune complexes in the glomerular basement membrane (108). Additionally, TLR4^+^CXCR4^+^ B cells have been implicated in driving the development of LN (109).

Reports indicate that more than half of LN patients exhibit abnormal infiltration of B cells in renal tissue (110). Abnormalities in ion channels are associated with the involvement of B cells in the pathogenesis of LN. B cells infiltrating the renal tissue of LN patients activate tissue adaptation programs in response to sodium stress, which upregulates Na^+^-K^+^-ATPase by renal epithelial cells, and moves three Na^+^ molecules extracellularly and two K^+^ molecules intracellularly against their concentration gradients. The process promotes the survival of pathologic B cells infiltrating the renal tissue under hyperosmolar conditions and aggravates the production of pathologic proteinuria (111). Inhibiting Na^+^ and K^+^ ATPase can reduce the number of infiltrating B cells and improve proteinuria levels in mouse models (111, 112). Using single-cell sequencing, researchers investigated the cell profile of renal biopsy tissue from lupus patients, enhancing our understanding of immune cell infiltration in LN and offering new insights into its pathogenesis, clinical diagnosis, and treatment. Tang et al. reported that the proportion of B cells in kidney tissue from LN patients reached 9.91%, compared to 6.79% in healthy controls. Additionally, the absolute quantity of B cells was significantly higher, with 84% in LN patients versus 16% in controls (113). Furthermore, Der et al. (114, 115) identified highly expressed interferon (IFN)-related genes in the renal parenchymal cells of LN patients through single-cell sequencing, however, they detected almost no B cells or lymphocytes in their study (116).

Additionally, we observed an increase in atypical memory B cells and long-lived plasma cells (LLPC), both of which play significant pathogenic roles in LN. Long-lived plasma cells infiltrate the renal tissue of LN patients, where they produce autoantibodies that form immune complexes, activating neutrophils and dendritic cells and ultimately leading to renal inflammation (117). Moreover, CD24^-^CD20^hi^ atypical memory B cells are significantly elevated in lupus patients and tend to aggregate in renal tissue, correlating with the severity of the disease (79). Single-cell sequencing of renal tissue from LN patients revealed that B cells also express high levels of age-associated markers. This characteristic differs from that of B cells found in the peripheral blood of these patients (44). The specific infiltration of B cells in renal tissue may drive an *in situ* immune response, contributing to tissue damage in lupus patients. Research indicates that the activated mTORC1 pathway is involved in the differentiation of atypical memory B cells. This differentiation enhances BCR activation and leads to metabolic dysregulation, which ultimately increases autoantibody secretion and promotes disease progression (79). Furthermore, ZEB2, a transcription factor, facilitates the formation of age-associated B cells (atypical memory B cells) (85). The infiltration of pathogenic B cells significantly contributes to local tissue damage in the kidneys of lupus patients. Depleting renal ABCs has been shown to alleviate lupus disease in the MRL/lpr lupus model (118).

### 3.5 B cell infiltration is involved in skin lesions of lupus patients

Approximately 70% of patients with SLE experience skin involvement, with about 20% presenting skin lesions as their initial manifestation. Skin lesions, such as butterfly-shaped erythema on the face, serve as specific indicators of lupus disease activity. In addition to these characteristic lesions, lupus patients may exhibit non-specific skin injuries, including thrombophlebitis, hair loss, Raynaud’s phenomenon, cutaneous ulceration, necrosis, and subungual splinter hemorrhages. Lupus can be classified into cutaneous lupus erythematosus and systemic lupus erythematosus based on the presence of affected organs beyond the skin (119). Lupus-related skin lesions are categorized into three types: acute cutaneous lupus erythematosus, subacute cutaneous lupus erythematosus, and chronic cutaneous lupus erythematosus (120). Notably, approximately 50% of cases of subacute cutaneous lupus erythematosus may progress to systemic lupus erythematosus, resulting in damage to multiple organs as the disease advances (120). Researchers primarily attribute these lesions to environmental factors, such as ultraviolet radiation, as well as imbalances in immune homeostasis. For instance, cytotoxic T cells may target keratinocyte apoptosis, while infiltrating neutrophils and plasma cell-like dendritic cells produce large amounts of interferon, contributing to the development of lupus-related cutaneous lesions (121).

In recent years, researchers have increasingly recognized the role of B cells in lupus-related cutaneous lesions. Reports indicate that lupus patients experience imbalanced B cell homeostasis; however, those with cutaneous lupus erythematosus display decreased levels of double negative B cells and increased levels of unswitched B cells compared to patients with systemic lupus erythematosus (122). Compared to healthy donors, lupus patients show significantly elevated expression of BAFF in both skin and serum. Specifically, the proportion of BAFF^+^ cells in the skin of lupus patients averages around 6.945 ± 1.386%, while in healthy donors, it is only 1.650 ± 0.884%. BAFF primarily localizes to the dermis, with minimal expression found in the epidermis (123). The infiltration of immune cells in the dermis and the activation of BAFF contribute to the pathological progression of lupus lesions.

In addition to BAFF, receptors such as BAFF-R, BCMA, and TACI are highly expressed in the skin of patients with subacute cutaneous lupus erythematosus and discoid lupus erythematosus, linking these receptors to pro-inflammatory mediators (124). Regarding the infiltration characteristics of different B cell subgroups, the average infiltration degree of CD20^+^ B cells is 17%. For BAFF-sensitive B cells, CD20^+^BAFF-R^+^ cells constitute 13%, while CD20^+^BAFF-R^+^MHC-II^+^ (antigen-presenting, BAFF-sensitive B cells) make up 12%. CD20^+^BAFF-R^+^MHC-II^+^CD80/86^+^ B cells (antigen-presenting, T cell-activated, BAFF-sensitive B cells) represent 1%. In terms of morphological characteristics, B cells can appear clustered or loosely arranged, and they may exhibit features of diffuse infiltration as individual cells (125).

Regarding the mechanisms of B cell involvement in lupus lesions, studies have shown that autoreactive B cells can be recruited into the tissue in response to inflammatory signals such as interferon, IL-21, and TLR7/9. This recruitment leads to the infiltration of CD20^+^ B cells and the formation of tertiary lymphoid structures in the skin, resulting in the secretion of large amounts of immunoglobulin and autoantibodies. These factors may further exacerbate local tissue inflammation and contribute to the progression of lupus (126–130). In a study involving 150 patients with lupus erythematosus, researchers observed that, compared to patients with subacute and acute cutaneous lupus erythematosus, those with discoid lupus erythematosus exhibited increased local infiltration and transcriptional activity of B cells in the tissue. Additionally, the infiltration of B cells, particularly memory B cells, in the skin lesions of patients with cutaneous lupus erythematosus was higher than that seen in patients with systemic lupus erythematosus. However, there was no significant difference in B cell infiltration in the skin of systemic lupus erythematosus patients compared to healthy donors. Interestingly, positive circulating autoantibodies, such as anti-nuclear antibodies and double-stranded DNA antibodies, in patients with cutaneous lupus did not correlate with B cell infiltration in skin lesions. These findings suggest that B cells exert pathogenic effects not only by secreting antibodies in skin lesions but also by producing antibodies that may not enter the circulation, indicating a strong organ heterogeneity in their function (131).

### 3.6 B cells and structural or functional brain damage in lupus patients

Neuropsychiatric lupus (NPSLE) is a complication involving the central nervous system in lupus patients, making it challenging to diagnose and treat. This condition primarily presents as headaches, anxiety, depression, and chronic cognitive dysfunction. Currently, the diagnosis of NPSLE relies mainly on scale assessments and brain magnetic resonance imaging (MRI). Scale assessments indicate that lupus patients experience a high incidence of anxiety and depression, alongside impaired cognitive function (132, 133). Imaging evaluations reveal compensatory reorganization of neural circuits in these patients, which may indicate adaptive or extensible neuroplasticity (134). Compared to healthy individuals, lupus patients exhibit abnormal blood flow perfusion in multiple brain regions, with significantly reduced average flow velocities observed in the hypothalamus, putamen, right posterior thalamus, and right anterior insula. Additionally, lupus patients show a marked increase in high-signal areas in the white matter related to perfusion indicators, such as cerebral blood flow, cerebral blood volume, and blood-brain barrier leakage parameters compared to normal white matter (135). The extensive leakage of the blood-brain barrier and the structural brain damage identified in lupus patients through imaging are significantly correlated with declines in cognitive function (136).

Due to the significant challenges in acquiring brain tissue from patients, current research on the mechanisms of central nervous system damage in lupus primarily focuses on analyzing cerebrospinal fluid and blood samples. Only a limited number of studies have reported pathological changes in the brain tissue of these patients (137, 138). Vascular lesions represent a key pathological feature of brain damage in lupus patients. These lesions include extensive micro-infarctions, lymphocytic perivascular infiltrates, atrophy, microbleeds, white matter hyperintensities, ischemic demyelination, and small subcortical infarcts (138, 139). An autopsy case report of a patient with NPSLE indicated that aggregates of white blood cells can lead to small vessel occlusions (140).

Studies using model animals indicate that B cells and autoantibodies can traverse the blood-brain barrier, promote an inflammatory microenvironment, and activate microglia. However, evidence supporting the presence of B cells in the central nervous system of lupus patients remains limited. In the cerebrospinal fluid and blood samples of lupus patients, high levels of cytokines such as BAFF and APRIL, along with the production of intrathecal IgG, are considered important indicators of B cell activation involved in brain tissue damage (141, 142). Injecting serum IgG from lupus patients into the brains of mice can induce microglial inflammation via the Fc signaling pathway. This finding suggests that elevated serum immunoglobulin levels may contribute to brain damage in lupus patients (143). In patients experiencing disease flares, particularly those with NPSLE, the expression of the chemokine receptor CXCR4 on B cells increases (144). Furthermore, the expression of brain-derived neurotrophic factor precursor (proBDNF) in peripheral antibody-secreting cells (ASCs, defined as CD19^+^CD27^hi^CD38^hi^) is significantly upregulated, and the presence of proBDNF^+^ ASCs closely correlates with disease activity in lupus patients (145). Dong et al. also reported a significant increase in ASCs in the peripheral blood of depressed lupus patients (13). Additionally, various autoantibodies, such as anti-NMDAR antibodies, anti-ribosomal P antibodies, and anti-phospholipid antibodies, are highly expressed in the cerebrospinal fluid of NPSLE patients (146). However, some studies have shown increased blood-brain barrier permeability, brain cell apoptosis, and high levels of cytokines in B cell-depleted mice. This suggests that B cells and autoantibodies may not be the primary factors leading to NPSLE (147). Overall, the scarcity of brain tissue samples from patients significantly limits our understanding of brain damage in lupus. Currently, there is no definitive conclusion regarding the role of B cells in the structural or functional damage to the brain in lupus patients.

### 3.7 B cells and cardiac, respiratory, ovarian gastrointestinal diseases, and arthritis in lupus patients

Cardiac involvement represents a significant complication in patients with lupus. These patients often experience vascular inflammation, where endothelial damage can promote atherosclerosis through the deposition of circulating immune complexes or the interaction of antibodies with endothelial cells. Endothelial dysfunction, along with the secretion of pro-inflammatory cytokines, can activate B cells, leading to the production of autoantibodies and the formation of immune complexes. This process further accelerates the intracellular accumulation of lipids in atherosclerotic plaques. Reports indicate that the carotid intima-media thickness in lupus patients is increased, indicating a susceptibility to subclinical atherosclerosis. This increase correlates with a decrease in the number of regulatory B cells (CD19^+^CD24^hi^CD38^hi^) (148). Immunohistochemical analyses of myocardial biopsy tissues from 14 lupus patients revealed deposits of immunoglobulin G and fibrinogen in the myocardial cell membrane and interstitium. These findings were accompanied by T cell infiltration, but no evidence of B cell infiltration was observed (149).

The lungs are also frequently affected in lupus patients, particularly in cases of interstitial lung disease. Among respiratory complications, diffuse alveolar hemorrhage is the most severe, with onset occurring within hours to days and a mortality rate as high as 50% (150). Treatment with CD19-targeting CAR-T or CD20-targeting rituximab has been shown to reduce levels of various autoantibodies and immunoglobulins, resulting in improvements in lung function (151–153). However, direct evidence demonstrating the involvement of B cells in the pathological mechanisms underlying lung injury in lupus patients is currently lacking.

Lupus is more prevalent in young women. Estrogen binding to B lymphocytes may trigger autoimmune reactions, resulting in the production of high-affinity autoantibodies and pro-inflammatory cytokines. This process can lead to estrogen-induced autoimmune diseases, such as autoimmune ovarian inflammation (154). Due to the combined effects of the disease and treatments like cyclophosphamide, lupus patients often experience reproductive organ dysfunction. This dysfunction can lead to decreased ovarian reserve, infertility, premature ovarian failure, and an increased risk of adverse pregnancy outcomes (155). Currently, there are no direct studies linking B cell abnormalities to ovarian dysfunction in lupus patients.

Regarding gastrointestinal diseases in lupus patients, research using animal models suggests that Helicobacter pylori infection can exacerbate inflammation, activate splenic B cells, and increase antibody secretion, ultimately promoting disease progression (156). An imbalance in gut microbiota is also suspected to contribute to the onset of lupus (157). Notably, the anaerobic bacteria from the Ruminococcus genus are found to be expanded in patients with active LN. Transplanting patient-derived strains into C57BL/6 mice leads to increased intestinal permeability, resulting in intestinal leakage and elevated levels of anti-dsDNA antibodies (158). Overall, research on the role of B cells in the gastrointestinal system of lupus patients remains limited.

Additionally, arthritis is one of the important organ disorders in SLE. However, current research on lupus B cells in articular tissue is still lacking, and further studies are needed to explore potential commonalities and distinctions between lupus and other joint diseases such as rheumatoid arthritis and osteoarthritis.

## 4 Clinical evaluation of B cell-targeted therapies

Current B cell-targeted therapies mainly include directly killing B cells, modulating B cell function, targeting molecular pathways for B cell growth and survival, and accelerating the degradation of autoantibodies. Traditional immunosuppressants are widely used in clinical practice, such as corticosteroids, hydroxychloroquine (HCQ), cyclophosphamide, mycophenolate mofetil, and azathioprine. There are also some drugs could inhibit B cell activation, proliferation, differentiation, and receptor signaling, such as Tripterygium wilfordii (159), SB431542 (160), rapamycin (161), KZR-616 (162), and Povetacicept (163). Additionally, biologic agents targeting B cells have emerged in recent years, as well as chimeric antigen receptor T cell (CAR-T) targeting the CD19 antigen, providing new hope for lupus patients (164).

Rituximab, a chimeric anti-CD20 monoclonal antibody, is used to deplete mature B cells and reduce the production of autoantibodies. About 98.6% patients exhibited depletion of peripheral blood CD19^+^B cells and reduction in serum levels of complement and anti-dsDNA antibody with rituximab treatment in LUNAR trial including 144 patients with LN of type III and type IV (165).

Belimumab, a fully recombinant humanized IgG2λ monoclonal antibody, is the first inhibitor targeting B-lymphocyte stimulator (BAFF/BLyS) (166). Belimumab reduced the number of immature B cells and CD11c^+^CD21^-^B cell at 3 months (167). After 76 weeks of treatment, significant reductions in B cell subpopulations were observed: naïve B cells, activated B cells, plasmacytoid cells, and plasma cells (168). Clinically, belimumab was effective in active lupus patients, who had a reduced risk of LN disease activity (169).

Telitacept, a human recombinant fusion targeting B lymphocyte stimulator, is a dual-target inhibitor of BAFF and APRIL (170). Telitacept could prevent the development and maturation of B cells by blocking BAFF, and reduce the number of CD19^+^ and IgD^+^ B cells in peripheral blood by blocking APRIL, decreasing the release of autoantibodies and immunoglobulin, and increasing complement levels (171, 172).

CD19-targeted CAR-T cell therapy may induce B cell depletion or co-target plasmablasts. Recently, researchers have been exploring CAR-T therapy targeting B cells in autoimmune diseases such as lupus (173). Some studies with small samples have produced great results for SLE patients and reported the CD19 CAR-T therapies in lupus appeared feasible, safe, efficacious and hopeful (174, 175).

The development of new drugs and therapies has brought significant progress, however, some patients still show unresponsive to treatment. Therefore, it is important to go further into exploring the characteristics of B cells in different organs or tissues.

## 5 Conclusion

**Figure 2:**
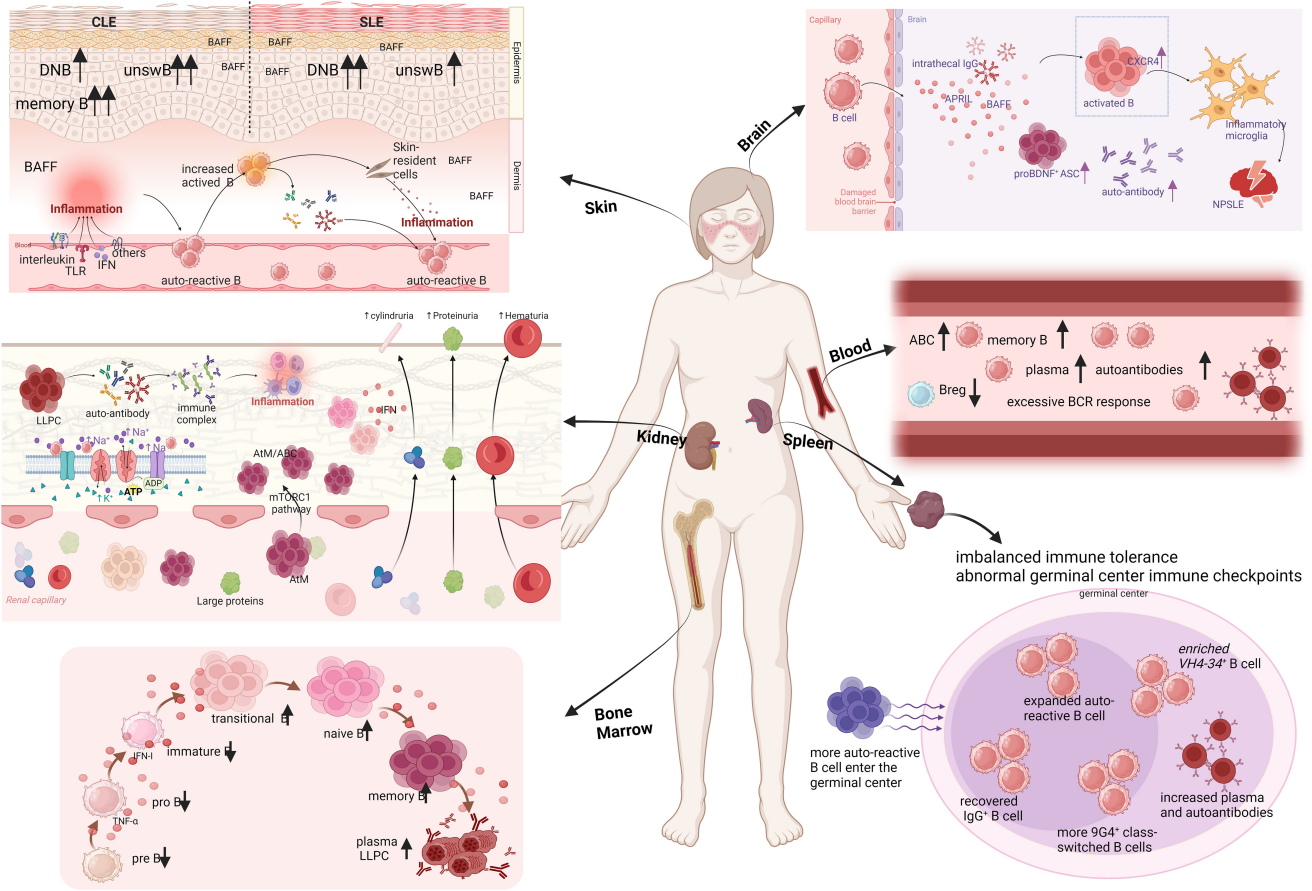
Organ-based characterization of B cells in lupus patients. CLE, cutaneous lupus erythematosus; SLE, systemic lupus erythematosus; DNB, double negative B cells; unswB, unswitched B cells; TLR, Toll-like receptors; IFN, interferon; ASC, antibody-secreting cells; NPSLE, neuropsychiatric lupus; LLPC, long-lived plasma cells; AtM, atypical memory B cells; ABC, age associated B cell (Created in BioRender. Dong, C. (2025) https://BioRender.com/o27s426).

The imbalance of B cell immune tolerance and the production of autoantibodies are central to the pathogenesis of lupus. However, the mechanisms by which immune tolerance is disrupted in lupus patients, the regulation of B cells during their development, differentiation, and maturation, and the targeted therapy of tissue-specific pathogenic B cells remain insufficiently understood. Disruption of peripheral immune tolerance, particularly involving immune checkpoints in the germinal center, is closely linked to the maturation of antibody affinity in autoreactive B cells. Additionally, B cells in the peripheral blood, kidney, skin, and bone marrow of lupus patients exhibit tissue-specific characteristics. For instance, pathogenic B cells in kidney tissue show high levels of infiltration and expression, primarily in the form of age-associated B cells. In contrast, skin tissue contains fewer disease-specific double-negative B cells, while bone marrow B cells demonstrate early developmental disorders, revealing significant abnormalities in their BCR repertoire during early development. Given these tissue-specific features, current strategies for targeted B cell therapy have yet to address these specific issues effectively (**Figure 2**). Through this review, we aim to offer new perspectives for future research related to B cells in lupus. This includes the development of targeted therapies and an important angle on the high heterogeneity of the disease.
